# Stool Samples of Acute Diarrhea Inpatients as a Reservoir of ST11 Hypervirulent KPC-2-Producing Klebsiella pneumoniae

**DOI:** 10.1128/mSystems.00498-20

**Published:** 2020-06-23

**Authors:** Beiwen Zheng, Hao Xu, Tao Lv, Lihua Guo, Yu Xiao, Chen Huang, Shuntian Zhang, Yunbo Chen, Huiming Han, Ping Shen, Yonghong Xiao, Lanjuan Li

**Affiliations:** a Collaborative Innovation Center for Diagnosis and Treatment of Infectious Diseases, State Key Laboratory for Diagnosis and Treatment of Infectious Diseases, the First Affiliated Hospital, College of Medicine, Zhejiang University, Hangzhou, China; b Basic Medical College, Beihua University, Jilin, China; c The Clinical Immunology Research Center, Beihua University, Jilin, China; University of California, San Francisco

**Keywords:** gut, hypervirulent, KPC-2, reservoir, genomic characterization

## Abstract

The emergence and spread of carbapenem-resistant hypervirulent Klebsiella pneumoniae sequence type 11 (ST11-CR-HvKP) in China are a great concern in the public health community. However, the underlying mechanism that enables its wide dissemination in China remains unclear. Here, we investigated the prevalence of carriage of carbapenemase-producing *Enterobacteriaceae* (CPE) among inpatients with diarrhea in a teaching hospital over 1 year to identify ST11-CR-HvKP reservoirs and to understand the genetic background and plasmid profiles of these pathogens. As assessed by stool analysis, the CPE colonization rate (12.4%) among the inpatients with diarrhea was high (12.4%). Antibiotic exposure, surgical history, and CPE positivity were correlated. Genomic investigation of 65 carbapenem-resistant K. pneumoniae isolates indicated a shared bacterial population in various wards. According to maximum likelihood phylogenetic tree analysis, these isolates were partitioned into three major clades. Analysis of the *wzi* locus revealed three different K types (KL105, KL47, and K64) among the ST11 isolates, indicating the genetic diversity of these isolates. Genetic and sequence mapping revealed the complexity of virulence and resistance plasmid sets harbored by the isolates. These observations indicate that the dissemination of resistant bacteria is more complex than initially anticipated and possibly involves multiple K. pneumoniae ST11 lineages and a variety of virulence plasmids. Collectively, we show for the first time that stool may be a source of ST11-CR-HvKP isolates. Furthermore, the findings reveal the silent dissemination of ST11-CR-HvKP bacteria in Zhejiang Province, China. Future investigations are warranted to determine the association between rectal colonization by ST11-CR-HvKP and clinical infections.

**IMPORTANCE** China has been experiencing a rapid increase in the number of nosocomial infections caused by carbapenem-resistant Klebsiella pneumoniae ST11 (ST11-CRKP) for decades. The emergence of hypervirulent ST11-CRKP (ST11-CR-HvKP) strains is expected to become a serious public health issue in China, considering that carbapenem resistance and virulence have converged in an epidemic clone. K. pneumoniae strains that colonize the human intestinal tract may become a reservoir of virulence and carbapenemase-encoding genes. Here, we first characterized the genotypes and antimicrobial phenotypes of ST11-CR-HvKP strains isolated from diarrheal stool samples of inpatients in Zhejiang Province, China. Active surveillance approaches based on the findings of the present study should be implemented, particularly in intensive care units, to combat the spread of ST11-CR-HvKP and to improve treatment.

## Abstract (precis)

China has been experiencing a rapid increase in the number of nosocomial infections caused by carbapenem-resistant Klebsiella pneumoniae ST11 (ST11-CRKP) for decades. The emergence of hypervirulent ST11-CRKP (ST11-CR-HvKP) strains is expected to become a serious public health issue in China, considering that carbapenem resistance and virulence have converged in an epidemic clone. K. pneumoniae strains that colonize the human intestinal tract may become a reservoir of virulence and carbapenemase-encoding genes. Here, we first characterized the genotypes and antimicrobial phenotypes of ST11-CR-HvKP strains isolated from diarrheal stool samples of inpatients in Zhejiang Province, China. Active surveillance approaches based on the findings of the present study should be implemented, particularly in intensive care units, to combat the spread of ST11-CR-HvKP and to improve treatment.

## INTRODUCTION

Carbapenem-resistant Klebsiella pneumoniae (CRKP), which is associated with high mortality rates of up to 50%, has been identified by the World Health Organization as a critical-priority organism (1, 2). Hypervirulent K. pneumoniae (HvKP) causes life-threatening infections, and the *rmpA* and *rmpA2* genes are associated with its pathogenicity (3). Recently, a fatal outbreak of sequence type 11 (ST11) carbapenem-resistant, hypervirulent K. pneumoniae (ST11-CR-HvKP) in China generated great concern from the public health community (4). The identification of this ST11-CR-HvKP strain demonstrated that carbapenem resistance and virulence had converged in an epidemic clone that could become a serious public health issue (5). Subsequently, cases of ST11-CR-HvKP infection have been documented in diverse regions in China (6–8). These reports suggest that this clone is widespread in China; however, the underlying mechanism that enables its wide dissemination remains unclear.

Intestinal tract colonization by carbapenemase-producing *Enterobacteriaceae* (CPE) may lead to a subsequent nosocomial infection in at-risk patients (9–11). We hypothesized that a relatively silent CPE gastrointestinal reservoir could exist in inpatients with diarrhea and could potentially be involved in the transmission of ST11-CR-HvKP. Accordingly, the aims of the present study were to investigate the prevalence of CPE carriage among inpatients with diarrhea in a teaching hospital in China over 1 year, to identify ST11-CR-HvKP reservoirs, and to explore the genomic complexity of the highly transmissible ST11-CR-HvKP clone in Zhejiang Province.

## RESULTS

During the study period, we sampled 811 nonduplicate stool samples from 443 inpatients with diarrhea and screened them for the presence of CPE strains. Ultimately, 87 CPE isolates from 55 patients were included in the study. This identification frequency indicated a high CPE colonization rate (i.e., 12.4% [55/443]) among these inpatients (see Fig. S1 in the supplemental material). Of these 87 isolates, K. pneumoniae was the most prevalent species (*n* = 65), followed by Proteus mirabilis (*n* = 6) and Escherichia coli (*n* = 6) (Table S1). PCR and sequencing revealed that 77 isolates carried *bla*_KPC-2_, 5 isolates carried *bla*_NDM-5_, 2 isolates carried *bla*_NDM-1_, 2 isolates carried *bla*_IMP-4_, and 1 isolate carried *bla*_IMP-26_. Furthermore, 44 isolates harbored extended-spectrum β-lactamase (ESBL) genes (Table S2). All CPE isolates were multidrug resistant (i.e., showed phenotypic resistance to three or more drug classes) (Fig. S2). Most of these CPE isolates were resistant to imipenem (99%) and susceptible to colistin (92%) and tigecycline (91%).

10.1128/mSystems.00498-20.1 FIG S1 Flowchart of the included cases and the analyses performed. Abbreviations: CPE, carbapenemase-producing *Enterobacteriaceae*; ARGs, antimicrobial resistance genes. Download FIG S1, TIF file, 1.9 MB. Copyright © 2020 Zheng et al. 2020 Zheng et al. This content is distributed under the terms of the Creative Commons Attribution 4.0 International license.

10.1128/mSystems.00498-20.2 FIG S2 Antimicrobial susceptibility profiles of 87 CPE isolates. The MICs were determined via an agar dilution method for all antibiotics except colistin and tigecycline, for which a broth microdilution method was used. Unless otherwise specified, the susceptibility tests were interpreted according to 2017 Clinical and Laboratory Standards Institute (CLSI) criteria. Pink indicates resistance, blue indicates intermediate, and yellow indicates sensitivity. Download FIG S2, TIF file, 1.6 MB. Copyright © 2020 Zheng et al. 2020 Zheng et al. This content is distributed under the terms of the Creative Commons Attribution 4.0 International license.

10.1128/mSystems.00498-20.5 TABLE S1 Carbapenemase-producing *Enterobacteriaceae* isolates recovered from stool specimens. Download Table S1, DOCX file, 0.02 MB. Copyright © 2020 Zheng et al. 2020 Zheng et al. This content is distributed under the terms of the Creative Commons Attribution 4.0 International license.

10.1128/mSystems.00498-20.6 TABLE S2 Summary of the carbapenemase-producing *Enterobacteriaceae* (CPE) isolates and the β-lactamases encoded by the CPE isolates. Download Table S2, DOCX file, 0.01 MB. Copyright © 2020 Zheng et al. 2020 Zheng et al. This content is distributed under the terms of the Creative Commons Attribution 4.0 International license.

Patients in medical intensive care units (ICUs) typically experience a prolonged hospital stay, which results in exposure to potential risk factors. Not surprisingly, therefore, most of the colonization cases were observed among patients from medical ICUs (Table S1). Colonization was detected in 13 wards: 49% (43/87) of cases were from the ICU, 9% were from the hepatobiliary and pancreatic surgery ward, 8% were from the emergency ICU, and the remaining 33% were from 10 other units. Forty-two (76%) of the inpatients were male, and the median inpatient age was 64 years (range, 16 to 97 years). Interestingly, correlations were detected between antibiotic exposure (including linezolid, a β-lactam–β-lactamase inhibitor combination, and carbapenems), surgical history, and being CPE positive (Table 1).

**TABLE 1:** Characteristics of the study participants and logistic regression analysis of the carbapenemase-producing *Enterobacteriaceae* strains isolated from stool samples from these inpatients

Variable	Value for group		*P*^*a*^	*P*^*b*^	OR (95% CI)
	CPE positive	CPE negative			
No. of patients (%)	55 (12)	388 (88)			
No. of male patients (%)	42 (76)	255 (66)	0.116	0.717	1.143 (0.556–2.350)
Median age (yr)^*c*^ (range)	64 (16–97)	55 (13–99)	0.047^*c*^	0.677	1.146 (0.603–2.179)
No. of patients (%) with:					
Surgery	16 (29)	54 (14)	0.004	0.047	2.078 (1.008–4.282)
Abdominal pain	13 (24)	130 (34)	0.14	0.578	0.818 (0.402–1.665)
Fever	43 (78)	244 (63)	0.026	0.595	1.257 (0.541–2.919)
No. of hospital-acquired infections (%)	32 (58)	199 (51)	0.124	0.958	0.97 (0.319–2.951)
No. receiving antibiotic therapy (%)					
Cephalosporins	15 (27)	73 (19)	0.141	0.349	1.4 (0.692–2.833)
Carbapenems	42 (76)	221 (57)	0.006	0.156	1.739 (0.81–3.732)
Cephamycin	0 (0)	15 (4)	0.234		
Aminoglycosides	5 (9)	38 (10)	0.869		
Macrolides	3 (6)	18 (5)	0.735		
Glycopeptides	20 (36)	108 (28)	0.192	0.674	1.151 (0.598–2.218)
Oral vancomycin	8 (15)	32 (8)	0.127	0.705	1.196 (0.473–3.021)
Fosfomycin	1 (2)	23 (6)	0.339		
Linezolid	23 (42)	56 (14)	<0.001	0.002	2.871 (1.461–5.642)
Quinolone	16 (29)	160 (41)	0.085	0.174	0.616 (0.306–1.238)
Sulfamethoxazole	4 (7)	68 (18)	0.054	0.457	0.637 (0.194–2.089)
β-Lactam–β-lactamase inhibitor	45 (82)	249 (64)	0.01	0.039	2.276 (1.043–4.969)
Tigecycline	15 (27)	63 (16)	0.044	0.67	0.849 (0.401–1.798)

a Univariate analysis; categorical variables were compared using a χ^2^ test.

b Multivariate analysis of variables (*P* ≤ 0.20) in the univariate analysis was performed using stepwise backward logistic regression. Odds ratios (ORs) and their 95% confidence intervals (CIs) were calculated.

c *P* values were calculated based on a comparison between the ages of ≥60 and <60 years using a χ^2^ test.

**FIG 1:**
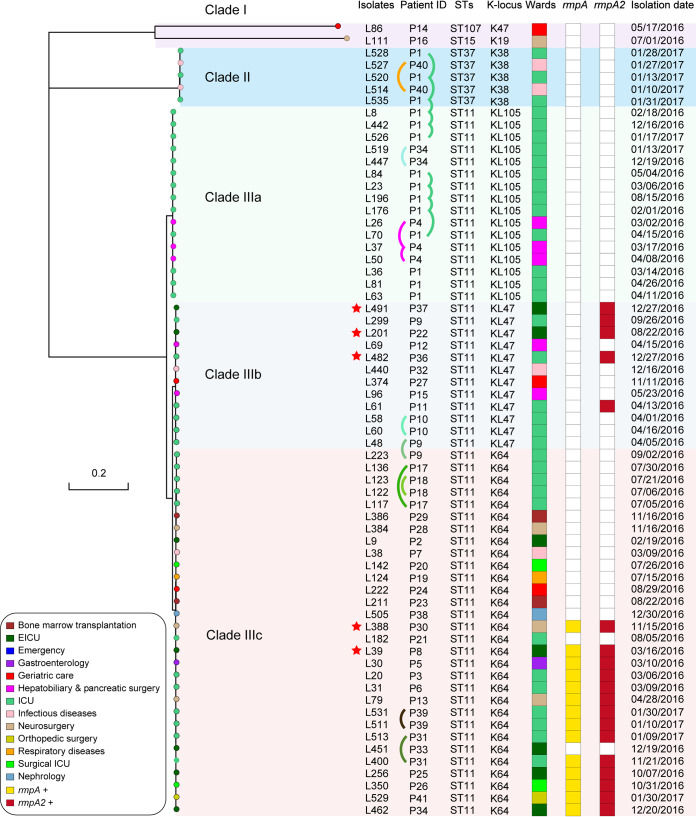
Core-genome phylogeny of the CRKP isolates, associated MLST data, capsule locus genotype, ward of isolation, virulence genes present, and isolation date. The maximum likelihood phylogeny tree is based on single-nucleotide polymorphisms in the core genomes of 65 CRKP isolates. The scale bar indicates nucleotide divergence. The origins of the isolates are shown in different colors. Genome clusters are shaded using different colors. The presence of the *rmpA* and *rmpA2* genes is indicated. The curved lines indicate isolates from the same individual. The red asterisks indicate the isolates that were analyzed by PacBio sequencing. EICU, emergency intensive care unit.

To better define the population structure of the 65 CRKP isolates, we further investigated their comprehensive molecular characteristics. Remarkably, multilocus sequence typing (MLST) analysis revealed that the K. pneumoniae isolates belonged to four STs, indicating low diversity (Fig. 1 and Table S1). ST11 was the predominant ST (58/65; 89%), followed by ST37 (*n* = 5), ST15 (*n* = 1), and ST107 (*n* = 1). A notable feature was that patient P1 (single individual) carried two STs, ST37, which was isolated from three different samples, and ST11, which was isolated from eight samples, over the entire study period. Furthermore, 10 patients in the study carried multiple isolates. Several major pulsotypes were found via pulsed-field gel electrophoresis (PFGE) analysis, which is consistent with the MLST results; however, genetic diversity was observed in the profiles (Fig. S3). Roary matrix-based gene sequence analysis generated a large pangenome of 10,355 gene clusters across 65 full genomes (Fig. S4). A maximum likelihood phylogenetic tree demonstrated that the 65 strains were partitioned into three major clades (Fig. 1). Seven non-ST11 isolates were clustered into two separate clades, and the 58 ST11 isolates were grouped into three clusters, despite their high similarity. An analysis of the *wzi* locus revealed three different K types (KL105, KL47, and K64) among the ST11 isolates, indicating genetic diversity among these isolates. Of note, ST11 isolates were detected in patients from 12 wards. A review of the cases revealed that these individuals had no contact with one another, lowering the probability of nosocomial transmission.

10.1128/mSystems.00498-20.3 FIG S3 PFGE dendrogram generated using BioNumerics software showing the genetic relationships among 65 CRKP isolates. PFGE pattern analysis demonstrated that 61 of the 65 CRKP isolates could be classified into nine pulsotypes: A (*n* = 2), B (*n* = 20), C (*n* = 9), D (*n* = 8), E (*n* = 8), F (*n* = 2), G (*n* = 2), H (*n* = 5), and I (*n* = 5). Four isolates appeared to be singletons. The dashed line corresponds to 80% as the cutoff for a close genetic relationship. Download FIG S3, TIF file, 1.4 MB. Copyright © 2020 Zheng et al. 2020 Zheng et al. This content is distributed under the terms of the Creative Commons Attribution 4.0 International license.

10.1128/mSystems.00498-20.4 FIG S4 Pangenome analysis of 65 CRKP strains, performed using Roary. The blue bar indicates the pangenome of CRKP, including the 10,355 annotated genes detected among the genomes analyzed in this study. Download FIG S4, TIF file, 2.0 MB. Copyright © 2020 Zheng et al. 2020 Zheng et al. This content is distributed under the terms of the Creative Commons Attribution 4.0 International license.

**FIG 2:**
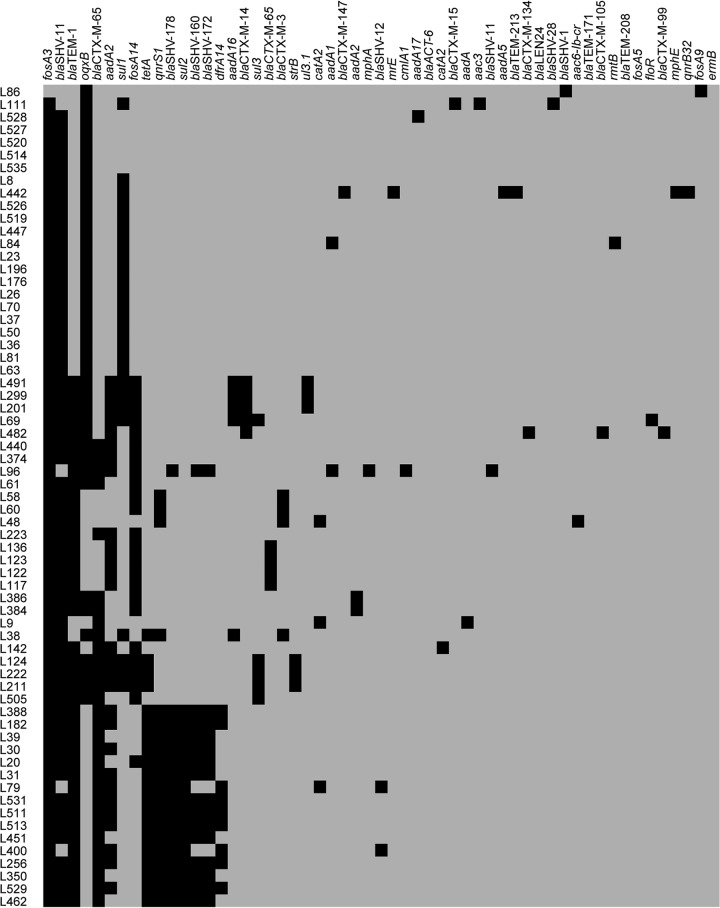
Antimicrobial resistance genes identified in the genomes of CRKP isolates by analyzing the WGS data. The resistance genes are shown at the top, with their presence indicated in black.

**FIG 3:**
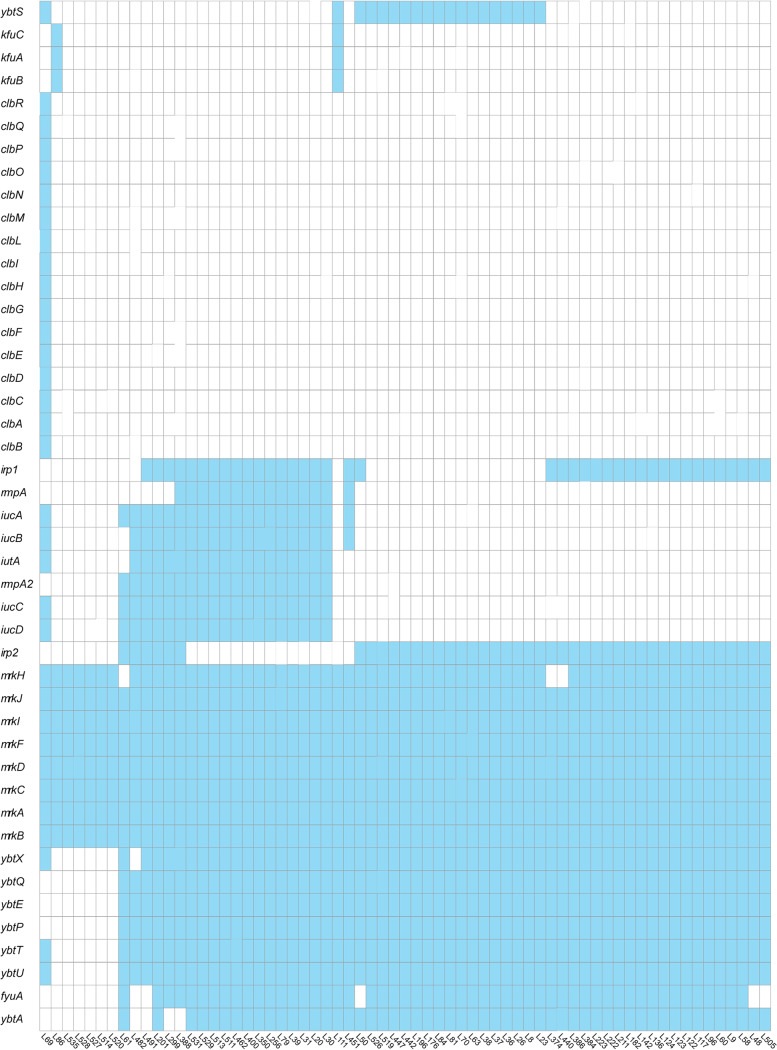
Distribution of virulence-associated genes in K. pneumoniae strains. Heat maps were generated by aligning the draft genome sequence of each isolate with the sequences deposited in the BIGSdb *Klebsiella* genome database. The presence of virulence genes in a specific genome is indicated by a blue box, and the absence of virulence genes is indicated by a white box. Virulence factors are shown at the left.

A broad array of resistance genes associated with various antimicrobials was identified in the K. pneumoniae genomes (Fig. 2). Isolates encoding carbapenemases (44/65; 68%) also harbored predicted ESBLs. Among these ESBLs, CTX-M-65 was the most predominant cluster (34/65; 52%), followed by CTX-M-14 (5/65; 8%). Virulence gene analysis revealed that the K. pneumoniae isolates carried genes for 45 known virulence factors (Fig. 3). As anticipated, the prevalence of biofilm-encoding type 3 fimbria cluster (*mrk*)- and yersiniabactin (*ybt*)-associated genes was high.

**FIG 4:**
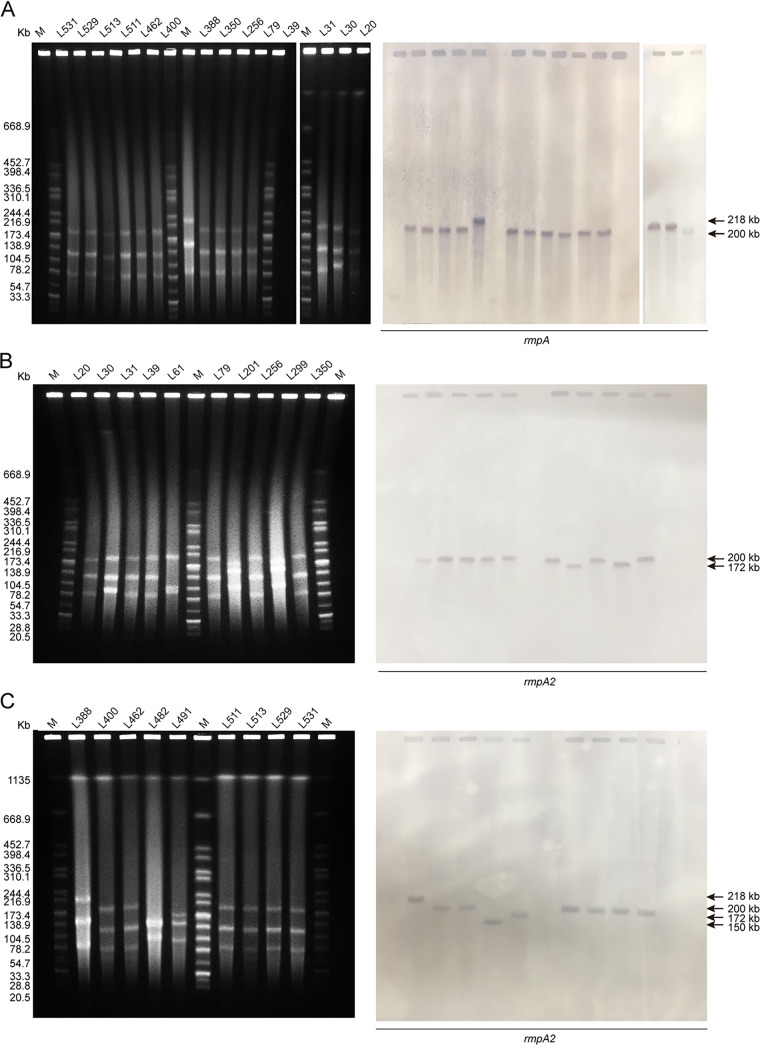
Analysis of plasmids harbored by the ST11-CR-HvKP isolates. The plasmids were analyzed by S1-PFGE and Southern blotting using specific *rmpA* and *rmpA2* probes. (A) Analysis of *rmpA*-carrying isolates. (B and C) Analysis of *rmpA2*-carrying isolates. The arrows indicate the locations of virulence plasmids. S1-PFGE revealed that most of the K. pneumoniae strains analyzed harbored three plasmids. M, marker.

**FIG 5:**
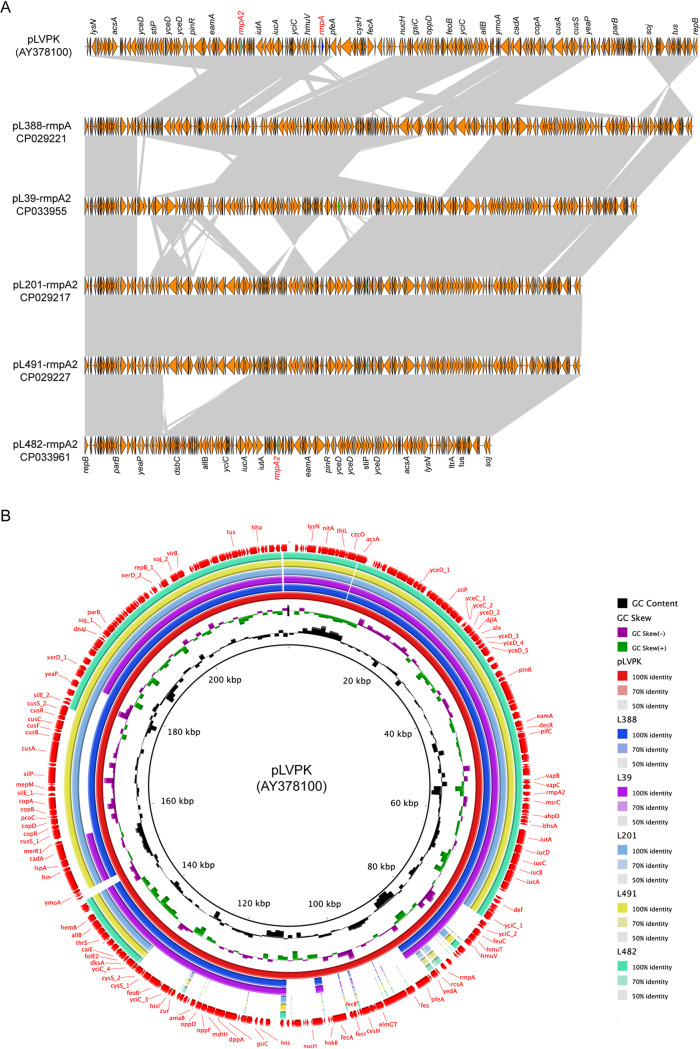
Alignment of the *rmpA*- and *rmpA2-*harboring plasmids recovered in the present study. The circular map was generated using the BLAST Ring Image Generator. (A) Colinear genome alignment of pLVPK (GenBank accession no. AY378100) with five *rmpA*- and *rmpA2-*harboring plasmids. (B) Genomic map of the *rmpA*- and *rmpA2*-harboring plasmids.

Using *in silico* analysis, we identified 14 *rmpA-* and 19 *rmpA2-*positive ST11-CRKP isolates. Interestingly, S1 nuclease PFGE (S1-PFGE) and Southern blot analyses clearly showed that the ST11-CR-HvKP isolates carried at least four types of *rmpA*- and *rmpA2*-positive plasmids, ranging in size from approximately 146 kb to approximately 218 kb (Fig. 4). The *rmpA* and *rmpA2* genes coexisted on the same plasmid in 14 isolates. We used Pacific Biosciences (PacBio) sequencing to generate five complete *rmpA*- and *rmpA2*-carrying IncHI1B plasmid sequences, which aligned well with the virulence plasmid pLVPK (GenBank accession no. AY378100), a 219-kb plasmid that harbors a set of virulence genes, including *iroBCDN*, *iucABCD*, *rmpA*, and *rmpA2* (Fig. 5).

## DISCUSSION

It is well recognized that intestinal tract colonization by Gram-negative bacteria may lead to a subsequent nosocomial infection in high-risk patients (12). In a recent large cohort study, Frencken et al. (13) demonstrated that rectal carriage of a Gram-negative pathogen is strongly associated with an increased risk of ICU-acquired bacteremia, suggesting that routine surveillance for gut colonization by Gram-negative bacteria may have clinical implications. Therefore, we designed the present study as a prospective survey to evaluate gut colonization by CPE and the associated risk factors among inpatients with diarrhea. We also performed an in-depth characterization of the gut flora CPE isolates.

ST11 is the most prevalent ST among CRKP isolates in China and accounts for up to 59.8% of CRKP isolates (14). We previously reported that 89.9% (124/138) of CRKP strains isolated from a university-affiliated hospital in 2014 carry the *bla*_KPC-2_ gene (15). Furthermore, K. pneumoniae ST11 undergoes rapid clonal expansion, resulting in regional outbreaks (16), which poses a challenge to patients, clinicians, and public health. The first ST11-CRKP strain in China was isolated at the First Affiliated Hospital of Zhejiang University (FAHZU) in 2004 (17). In the past few decades, China has witnessed the emergence and subsequent rapid increase in the number of ST11-CRKP nosocomial infections (14). HvKP is rarely resistant to antibiotics. The occurrence of ST11-CR-HvKP threatens the effective management of infections caused by this clinically untreatable pathogen, which could become a serious public health problem and even cause a global CR-HvKP epidemic (5). However, the role of the gut microbiota in the rapid spread of ST11-CR-HvKP has not yet been elucidated. Experimental evidence from the present study, as well as from retrieved clinical data, indicated a disturbing rate of carriage of ST11-CRKP (8.6%) among hospitalized patients with acute diarrhea, particularly in ICU wards. Furthermore, the findings of the present study suggest that stool samples could be a major reservoir of ST11-CR-HvKP. Active surveillance of nondiarrheal patients is required to compare their rates of CPE colonization with those of diarrheal patients and to investigate whether the implementation of contact precautions or other interventions would improve outcomes.

Not surprisingly, in the present study, the majority of colonization cases were observed among patients in the medical ICU. Recently, a systematic review of the clinical epidemiology of CRE identiﬁed 13 risk factors associated with CRE acquisition (18). These are medical devices, carbapenem use, invasive procedures, ICU admission, cephalosporin use, glycopeptide use, CRE exposure, underlying diseases, quinolone use, β-lactam use, mechanical ventilation, demographic patient characteristics, and exposure to hospital care. The findings of the present study are consistent with those observations, although we focused on gut colonization by CPE and not on CPE-associated infections. To the best of our knowledge, an association between linezolid exposure and CPE colonization in patients has not been reported to date. The lack of such an association might be explained by the wide use of linezolid, which eliminates Gram-positive bacteria and disrupts the gut microflora, thus promoting gut colonization by *Enterobacteriaceae*, especially by CPE strains.

A previous investigation speculated that the emergence of ST11-CR-HvKP occurred due to a single genetic event in which a pLVPK-like virulence plasmid was acquired by an ST11-CRKP strain that presumably carried a common plasmid such as pKPC-CR-HvKP4 (4). The observed complexity in the existence of virulence plasmids and resistance plasmids in the ST11-CRKP isolates indicates that this process was more complicated than was previously anticipated, as it may have involved multiple ST11 K. pneumoniae lineages and a variety of virulence plasmids.

This study has several limitations. First, our study is limited by its single-institution design and 1-year study period; therefore, the findings of this study should be interpreted with caution and might not be generalizable to other hospitals. Second, although we identified 19 fecal ST11-CR-HvKP colonization isolates, none of the colonized patients had ST11-CR-HvKP infections. Third, the findings probably reflect the status of diarrheal patients, i.e., individuals with a disrupted gut microbiota. A previous investigation described the high prevalence and mortality rate of ST11-CR-HvKP meningitis in FAHZU (8), and K64 was also the most common serotype detected in our study. Future investigations addressing the transition from ST11-CR-HvKP carriage to infection in high-risk patients are crucial for the management of such infections.

In conclusion, our genomic epidemiological investigation demonstrated that stool samples of hospitalized patients served as a reservoir of CPE isolates, particularly ST11-CR-HvKP. Because of the serious clinical outcomes of CRKP infections, more information is needed to understand the potential risk of ST11-CR-HvKP spreading by inpatients and to develop measures for the surveillance or control of these risks.

## MATERIALS AND METHODS

### Study design.

A prospective, observational cohort study involving inpatients with diarrhea was performed between February 2016 and February 2017 (see Fig. S1 in the supplemental material) at the First Affiliated Hospital of Zhejiang University (FAHZU), China. FAHZU is the largest tertiary teaching hospital in Zhejiang Province, with 2,500 beds. *Enterobacteriaceae* isolates cultured from the first collected clinical samples of the respective inpatients were collected. Duplicate isolates (the same species isolated from material from the same patient within 2 weeks of the first positive culture) were excluded. All CPE isolates underwent antimicrobial susceptibility testing, molecular typing, and whole-genome sequencing (WGS). Clinical data for all participants were extracted from the clinical and medical record system using centralized queries. Information on demographic data, characteristics at admission, surgical intervention, hospital-acquired infections, and antibiotic exposure during the hospital stay were collected for CPE carriers and noncarriers.

### Bacterial isolation and identification.

Fecal samples (1.0 g) were diluted in 5 ml of sterile Luria-Bertani broth and cultured overnight at 37°C. The cultures grown overnight were plated on MacConkey agar supplemented with 1 mg/liter meropenem for 18 to 24 h at 37°C to isolate potential carbapenem-resistant *Enterobacteriaceae* (CRE) strains. Colonies with different morphologies were repeatedly streaked on MacConkey agar with meropenem to obtain pure isolates. Bacterial species were identified by matrix-assisted laser desorption ionization–time of flight mass spectrometry and 16S rRNA gene sequencing using universal prokaryotic primers. CRE isolates were confirmed by susceptibility testing in the presence of imipenem, ertapenem, and meropenem via a broth microdilution method (19). Genomic DNA was extracted from cultures grown overnight using the Qiagen (Hilden, Germany) blood/tissue kit. CPE isolates were identified by detecting carbapenemase-encoding genes (*bla*_NDM_, *bla*_KPC_, *bla*_IMP_, and *bla*_VIM_). The genes were amplified by PCR, with 1 μl of genomic DNA from each isolate as a template, and the nature of the amplicons was then confirmed by sequencing (20).

### Characterization of HvKP.

The presence of the virulence-associated genes *rmpA* (regulator of mucoid phenotype A), *rmpA2* (activator for capsule biosynthesis), *iucA*, and *iutA* (21, 22) was examined in HvKP strains by PCR. The PCR products were first visualized by 1% agarose gel electrophoresis, and the nature of the amplicons was then confirmed by direct Sanger sequencing. Furthermore, the hypermucoviscous phenotype was analyzed by using the string test, as described elsewhere (23). The cutoff criterion for positivity in the string test was a viscous string length of >5 mm.

### Risk factor analysis.

To investigate the risk factors associated with fecal carriage of CPE, carriers were compared with noncarriers in terms of exposure to different variables. Categorical variables were expressed as percentages and compared using the chi-squared test or two-tailed Fisher exact test, as appropriate. Independent predictors for CPE were examined by logistic regression analysis. Variables with a *P* value of ≤0.2 (univariate analysis) were included in a logistic regression model to identify the variables with either a negative or a positive impact on the CPE colonization of inpatients with acute diarrhea. The strength of associations was determined by calculating the odds ratios (ORs) and 95% confidence intervals (CIs). Variables with a two-tailed *P* value of <0.05 were considered statistically significant. All statistical analyses were performed using SPSS version 24.0.

### Antimicrobial susceptibility testing.

MIC values of 14 antimicrobial agents (amikacin, aztreonam, cefotaxime, cefpirome, ceftazidime, ciprofloxacin, colistin, fosfomycin, gentamicin, imipenem, meropenem, piperacillin-tazobactam, tobramycin, and tigecycline) were determined by an agar dilution method, except for colistin and tigecycline, for which the broth microdilution method was used. The results were interpreted using Clinical and Laboratory Standards Institute guideline breakpoints (19). Standard reference strains of Escherichia coli, ATCC 25922, and K. pneumoniae, ATCC 700603, were used for quality control.

### Molecular typing of KPC-2-producing K. pneumoniae isolates.

The genetic relatedness of K. pneumoniae isolates was assessed by pulsed-field gel electrophoresis (PFGE). Briefly, DNA fragments were separated by 1% agarose gel electrophoresis (SeaKem gold agarose; Lonza, USA) at 14°C and 6 V/cm and with an alternating pulse at 120°, with a 2- to 40-s pulse-time gradient, for 22 h in 0.5× Tris-boric acid-EDTA buffer using a Chef apparatus (Bio-Rad, USA). Salmonella enterica serotype Braenderup H9812 was used as a size marker (24). The PFGE pattern dendrogram was constructed using BioNumerics version 7.6 (Applied Maths, Sint-Martens-Latem, Belgium) with UPGMA (unweighted pair group method using average linkages) clustering. Isolates with a similarity cutoff of ≥80% were considered to be a pulsotype.

### Illumina sequencing and sequence assembly.

To characterize the genetic features and resistome of KPC-2-producing K. pneumoniae isolates, WGS was performed. Genomic DNA was extracted from cultures grown overnight using the Gentra Puregene Yeast/Bact kit (Qiagen). The extracted DNA was evaluated by 1% (wt/vol) agarose gel electrophoresis, and its concentration and purity were determined using a NanoDrop 2000 UV-visible (UV-Vis) spectrophotometer (Thermo Scientific, Waltham, MA, USA) and a Qubit version 2.0 fluorometer (Thermo Scientific). DNA was stored at −20°C until further processing. The sequencing library was prepared by using a Nextera XT kit (Illumina, San Diego, CA, USA) and sequenced using the Illumina HiSeq X 10-PE150 platform (Illumina). A-tailed fragments were ligated with paired-end adaptors and PCR amplified with a 500-bp insert. A mate pair library with an insert size of 5 kb was used for library construction by Beijing Novogene Bioinformatics Technology Co., Ltd. PCR adaptor reads and low-quality reads from the paired-end and mate pair library were filtered during a quality control step using the Novogene pipeline. Paired reads were then assembled into scaffolds using Velvet version 1.2.10 (25).

### Long-read Pacific Biosciences sequencing and assembly.

To elucidate the genetic environment of K. pneumoniae isolates carrying the *rmpA* and *rmpA2* genes, five *rmpA*- and *rmpA2*-positive isolates were selected based on the size of the *rmpA*- and *rpmA2*-carrying plasmids and analyzed by PacBio sequencing. DNA extracted from isolates L39, L201, L388, L482, and L491 was sequenced by using long-read single-molecule real-time (SMRT) sequencing technology and the PacBio (Menlo Park, CA, USA) RS II platform. Briefly, the extracted high-quality double-stranded DNA (10 μg) was sequenced using P6-C4 chemistry, PacBio RS II instrumentation, and a complexed 20-kb SMRTbell library. Unicycler was used for a hybrid assembly of three bacterial genomes from a combination of Illumina short reads and PacBio long reads (26). To generate the best assembly, Unicycler was operated in three different modes (conservative, normal, and bold) that alter the cutoff for a minimum acceptable bridge quality.

### Bioinformatics analysis and phylogenomic computations.

To determine the clonal lineages, the sequence types (STs) of KPC-2-producing K. pneumoniae isolates were determined by multilocus sequence typing (MLST) of WGS data, in accordance with protocols described on the Institut Pasteur website (http://bigsdb.pasteur.fr/klebsiella/). ResFinder version 2.1 (http://cge.cbs.dtu.dk/services/resfinder) was used to identify antimicrobial resistance genes. Plasmid Finder version 1.3 was used to identify the plasmid incompatibility type (27). The virulence loci in the assembled genome sequences were identified using the BIGSdb *Klebsiella* genome database. A heat map of virulence loci was generated using Genesis software version 1.7.7. The core genes in the genomes of KPC-2-producing K. pneumoniae strains were identified using Prokka (28) and Roary (29). Maximum likelihood-based phylogenetic reconstruction was performed with RAxML version 8.2.10 using the generalized time-reversible (GTR) + Γ nucleotide substitution model (30). One hundred bootstrap replicates were evaluated to determine branch support. A maximum likelihood phylogenetic tree based on the core single-nucleotide polymorphism alignments was generated using FastTree (31).

### Characterization of the *rmpA*- and *rmpA2-*carrying plasmids.

S1 nuclease pulsed-field gel electrophoresis (S1-PFGE) and Southern blot analysis were performed to estimate the size of the *rmpA*- and *rmpA2*-carrying plasmids (32). Plasmid sequences were assembled from WGS data using plasmidSPAdes (33) and annotated using the RAST tool (34). The sequences of representative plasmids were compared against plasmid sequences deposited in the National Center for Biotechnology Information database using BLAST and plotted by using the BLAST Ring Image Generator (35).

### Data availability.

The complete whole-genome sequences of 65 CRKP isolates have been deposited in the GenBank database under BioProject accession no. PRJNA390758.

## References

[1] Giacobbe DR, Del Bono V, Trecarichi EM, De Rosa FG, Giannella M, Bassetti M, Bartoloni A, Losito AR, Corcione S, Bartoletti M, Mantengoli E, Saffioti C, Pagani N, Tedeschi S, Spanu T, Rossolini GM, Marchese A, Ambretti S, Cauda R, Viale P, Viscoli C, Tumbarello M, Isgri S, Italian Study Group on Resistant Infections of the Società Italiana Terapia Antinfettiva. 2015 Risk factors for bloodstream infections due to colistin-resistant KPC-producing Klebsiella pneumoniae: results from a multicenter case-control-control study. Clin Microbiol Infect 21:1106.e1–1106.e8. doi:10.1016/j.cmi.2015.08.001. 26278669

[2] Pecora ND, Li N, Allard M, Li C, Albano E, Delaney M, Dubois A, Onderdonk AB, Bry L 2015 Genomically informed surveillance for carbapenem-resistant Enterobacteriaceae in a health care system. mBio 6:e01030-15. doi:10.1128/mBio.01030-15. 26220969 PMC4551976

[3] Russo TA, Marr CM 2019 Hypervirulent Klebsiella pneumoniae. Clin Microbiol Rev 32:e00001-19. doi:10.1128/CMR.00001-19. 31092506 PMC6589860

[4] Gu D, Dong N, Zheng Z, Lin D, Huang M, Wang L, Chan EW, Shu L, Yu J, Zhang R, Chen S 2018 A fatal outbreak of ST11 carbapenem-resistant hypervirulent Klebsiella pneumoniae in a Chinese hospital: a molecular epidemiological study. Lancet Infect Dis 18:37–46. doi:10.1016/S1473-3099(17)30489-9. 28864030

[5] Chen L, Kreiswirth BN 2018 Convergence of carbapenem-resistance and hypervirulence in Klebsiella pneumoniae. Lancet Infect Dis 18:2–3. doi:10.1016/S1473-3099(17)30517-0. 28864028

[6] Wong MHY, Shum H-P, Chen JHK, Man M-Y, Wu A, Chan EW-C, Yuen K-Y, Chen S 2018 Emergence of carbapenem-resistant hypervirulent Klebsiella pneumoniae. Lancet Infect Dis 18:24. doi:10.1016/S1473-3099(17)30629-1. 29102517

[7] Yao H, Qin S, Chen S, Shen J, Du XD 2018 Emergence of carbapenem-resistant hypervirulent Klebsiella pneumoniae. Lancet Infect Dis 18:25. doi:10.1016/S1473-3099(17)30628-X. 29102518

[8] Xu M, Fu Y, Fang Y, Xu H, Kong H, Liu Y, Chen Y, Li L 2019 High prevalence of KPC-2-producing hypervirulent Klebsiella pneumoniae causing meningitis in Eastern China. Infect Drug Resist 12:641–653. doi:10.2147/IDR.S191892. 30936727 PMC6430001

[9] Viau R, Frank KM, Jacobs MR, Wilson B, Kaye K, Donskey CJ, Perez F, Endimiani A, Bonomo RA 2016 Intestinal carriage of carbapenemase-producing organisms: current status of surveillance methods. Clin Microbiol Rev 29:1–27. doi:10.1128/CMR.00108-14. 26511484 PMC4771221

[10] Gorrie CL, Mirceta M, Wick RR, Edwards DJ, Thomson NR, Strugnell RA, Pratt NF, Garlick JS, Watson KM, Pilcher DV, McGloughlin SA, Spelman DW, Jenney AWJ, Holt KE 2017 Gastrointestinal carriage is a major reservoir of Klebsiella pneumoniae infection in intensive care patients. Clin Infect Dis 65:208–215. doi:10.1093/cid/cix270. 28369261 PMC5850561

[11] Shimasaki T, Seekatz A, Bassis C, Rhee Y, Yelin RD, Fogg L, Dangana T, Cisneros EC, Weinstein RA, Okamoto K, Lolans K, Schoeny M, Lin MY, Moore NM, Young VB, Hayden MK, Centers for Disease Control and Prevention Epicenters Program. 2019 Increased relative abundance of Klebsiella pneumoniae carbapenemase-producing Klebsiella pneumoniae within the gut microbiota is associated with risk of bloodstream infection in long-term acute care hospital patients. Clin Infect Dis 68:2053–2059. doi:10.1093/cid/ciy796. 30239622 PMC6541703

[12] Sertaridou E, Papaioannou V, Kolios G, Pneumatikos I 2015 Gut failure in critical care: old school versus new school. Ann Gastroenterol 28:309–322. 26130136 PMC4480167

[13] Frencken JF, Wittekamp BHJ, Plantinga NL, Spitoni C, van de Groep K, Cremer OL, Bonten MJM 2018 Associations between enteral colonization with Gram-negative bacteria and intensive care unit-acquired infections and colonization of the respiratory tract. Clin Infect Dis 66:497–503. doi:10.1093/cid/cix824. 29186403

[14] Zhang R, Liu L, Zhou H, Chan EW, Li J, Fang Y, Li Y, Liao K, Chen S 2017 Nationwide surveillance of clinical carbapenem-resistant Enterobacteriaceae (CRE) strains in China. EBioMedicine 19:98–106. doi:10.1016/j.ebiom.2017.04.032. 28479289 PMC5440625

[15] Lv J, Zheng B, Zhang J, Yu W, Dong H, Li LJ, Xiao Y 2016 Molecular characterization of carbapenem-resistant Klebsiella pneumoniae collected from a teaching hospital. Chin J Antibiot 41:356–361.

[16] Qi Y, Wei Z, Ji S, Du X, Shen P, Yu Y 2011 ST11, the dominant clone of KPC-producing Klebsiella pneumoniae in China. J Antimicrob Chemother 66:307–312. doi:10.1093/jac/dkq431. 21131324

[17] Wei Z-Q, Du X-X, Yu Y-S, Shen P, Chen Y-G, Li L-J 2007 Plasmid-mediated KPC-2 in a Klebsiella pneumoniae isolate from China. Antimicrob Agents Chemother 51:763–765. doi:10.1128/AAC.01053-06. 17145797 PMC1797727

[18] van Loon K, Voor in ‘t Holt AF, Vos MC 2018 A systematic review and meta-analyses of the clinical epidemiology of carbapenem-resistant Enterobacteriaceae. Antimicrob Agents Chemother 62:e01730-17. doi:10.1128/AAC.01730-17. 29038269 PMC5740327

[19] CLSI. 2017 Performance standards for antimicrobial susceptibility testing, 27th ed CLSI supplement M100 CLSI, Wayne, PA.

[20] Zheng B, Zhang J, Ji J, Fang Y, Shen P, Ying C, Lv J, Xiao Y, Li L 2015 Emergence of Raoultella ornithinolytica coproducing IMP-4 and KPC-2 carbapenemases in China. Antimicrob Agents Chemother 59:7086–7089. doi:10.1128/AAC.01363-15. 26282422 PMC4604418

[21] Choi MJ, Ko KS 2015 Loss of hypermucoviscosity and increased fitness cost in colistin-resistant Klebsiella pneumoniae sequence type 23 strains. Antimicrob Agents Chemother 59:6763–6773. doi:10.1128/AAC.00952-15. 26282408 PMC4604379

[22] Zhang Y, Jin L, Ouyang P, Wang Q, Wang R, Wang J, Gao H, Wang X, Wang H, China Carbapenem-Resistant Enterobacteriaceae Network. 2020 Evolution of hypervirulence in carbapenem-resistant Klebsiella pneumoniae in China: a multicentre, molecular epidemiological analysis. J Antimicrob Chemother 75:327–336. doi:10.1093/jac/dkz446. 31713615

[23] Cubero M, Grau I, Tubau F, Pallares R, Dominguez MA, Linares J, Ardanuy C 2016 Hypervirulent Klebsiella pneumoniae clones causing bacteraemia in adults in a teaching hospital in Barcelona, Spain (2007-2013). Clin Microbiol Infect 22:154–160. doi:10.1016/j.cmi.2015.09.025. 26454059

[24] Shen P, Wei Z, Jiang Y, Du X, Ji S, Yu Y, Li L 2009 Novel genetic environment of the carbapenem-hydrolyzing beta-lactamase KPC-2 among Enterobacteriaceae in China. Antimicrob Agents Chemother 53:4333–4338. doi:10.1128/AAC.00260-09. 19620332 PMC2764158

[25] Zerbino DR, Birney E 2008 Velvet: algorithms for de novo short read assembly using de Bruijn graphs. Genome Res 18:821–829. doi:10.1101/gr.074492.107. 18349386 PMC2336801

[26] Wick RR, Judd LM, Gorrie CL, Holt KE 2017 Unicycler: resolving bacterial genome assemblies from short and long sequencing reads. PLoS Comput Biol 13:e1005595. doi:10.1371/journal.pcbi.1005595. 28594827 PMC5481147

[27] Carattoli A, Zankari E, García-Fernández A, Voldby Larsen M, Lund O, Villa L, Møller Aarestrup F, Hasman H 2014 In silico detection and typing of plasmids using PlasmidFinder and plasmid multilocus sequence typing. Antimicrob Agents Chemother 58:3895–3903. doi:10.1128/AAC.02412-14. 24777092 PMC4068535

[28] Seemann T 2014 Prokka: rapid prokaryotic genome annotation. Bioinformatics 30:2068–2069. doi:10.1093/bioinformatics/btu153. 24642063

[29] Page AJ, Cummins CA, Hunt M, Wong VK, Reuter S, Holden MT, Fookes M, Falush D, Keane JA, Parkhill J 2015 Roary: rapid large-scale prokaryote pan genome analysis. Bioinformatics 31:3691–3693. doi:10.1093/bioinformatics/btv421. 26198102 PMC4817141

[30] Stamatakis A 2014 RAxML version 8: a tool for phylogenetic analysis and post-analysis of large phylogenies. Bioinformatics 30:1312–1313. doi:10.1093/bioinformatics/btu033. 24451623 PMC3998144

[31] Price MN, Dehal PS, Arkin AP 2009 FastTree: computing large minimum evolution trees with profiles instead of a distance matrix. Mol Biol Evol 26:1641–1650. doi:10.1093/molbev/msp077. 19377059 PMC2693737

[32] Zheng B, Dong H, Xu H, Lv J, Zhang J, Jiang X, Du Y, Xiao Y, Li L 2016 Coexistence of MCR-1 and NDM-1 in clinical Escherichia coli isolates. Clin Infect Dis 63:1393–1395. doi:10.1093/cid/ciw553. 27506685

[33] Antipov D, Hartwick N, Shen M, Raiko M, Lapidus A, Pevzner PA 2016 plasmidSPAdes: assembling plasmids from whole genome sequencing data. Bioinformatics 32:3380–3387. doi:10.1093/bioinformatics/btw493. 27466620

[34] Overbeek R, Olson R, Pusch GD, Olsen GJ, Davis JJ, Disz T, Edwards RA, Gerdes S, Parrello B, Shukla M, Vonstein V, Wattam AR, Xia F, Stevens R 2014 The SEED and the Rapid Annotation of microbial genomes using Subsystems Technology (RAST). Nucleic Acids Res 42:D206–D214. doi:10.1093/nar/gkt1226. 24293654 PMC3965101

[35] Alikhan NF, Petty NK, Ben Zakour NL, Beatson SA 2011 BLAST Ring Image Generator (BRIG): simple prokaryote genome comparisons. BMC Genomics 12:402. doi:10.1186/1471-2164-12-402. 21824423 PMC3163573

