# Supplementary figures and images for: Stool Samples of Acute Diarrhea Inpatients as a Reservoir of ST11 Hypervirulent KPC-2-Producing Klebsiella pneumoniae
Source: mSystems. 2020 Jun 23;5(3):e00498-20. doi: 10.1128/mSystems.00498-20 (PMC7311318; doi:10.1128/mSystems.00498-20)

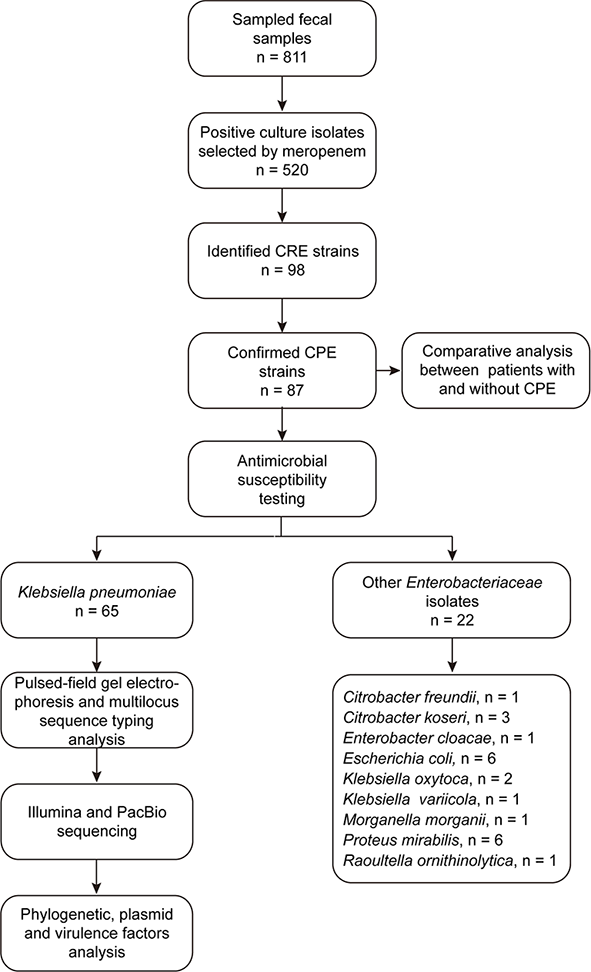

Supplement: FIG S1 [file mSystems.00498-20-sf001.tif]

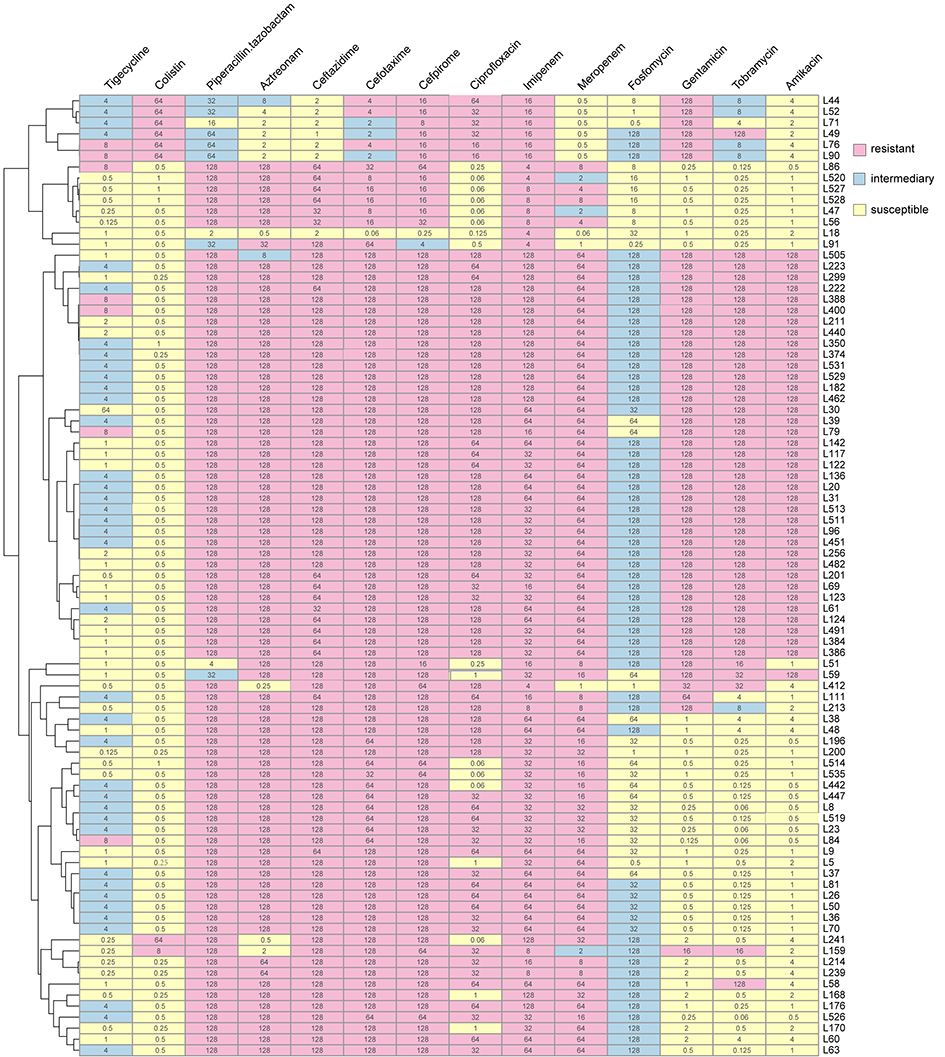

Supplement: FIG S2 [file mSystems.00498-20-sf002.tif]

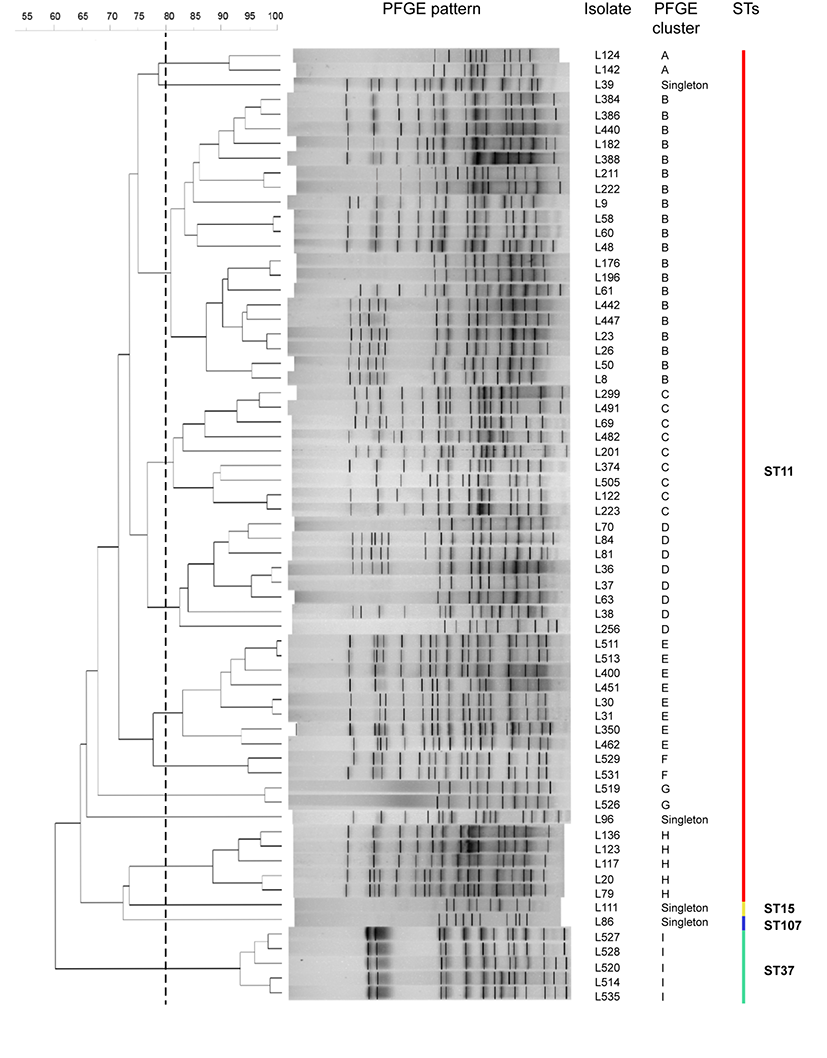

Supplement: FIG S3 [file mSystems.00498-20-sf003.tif]

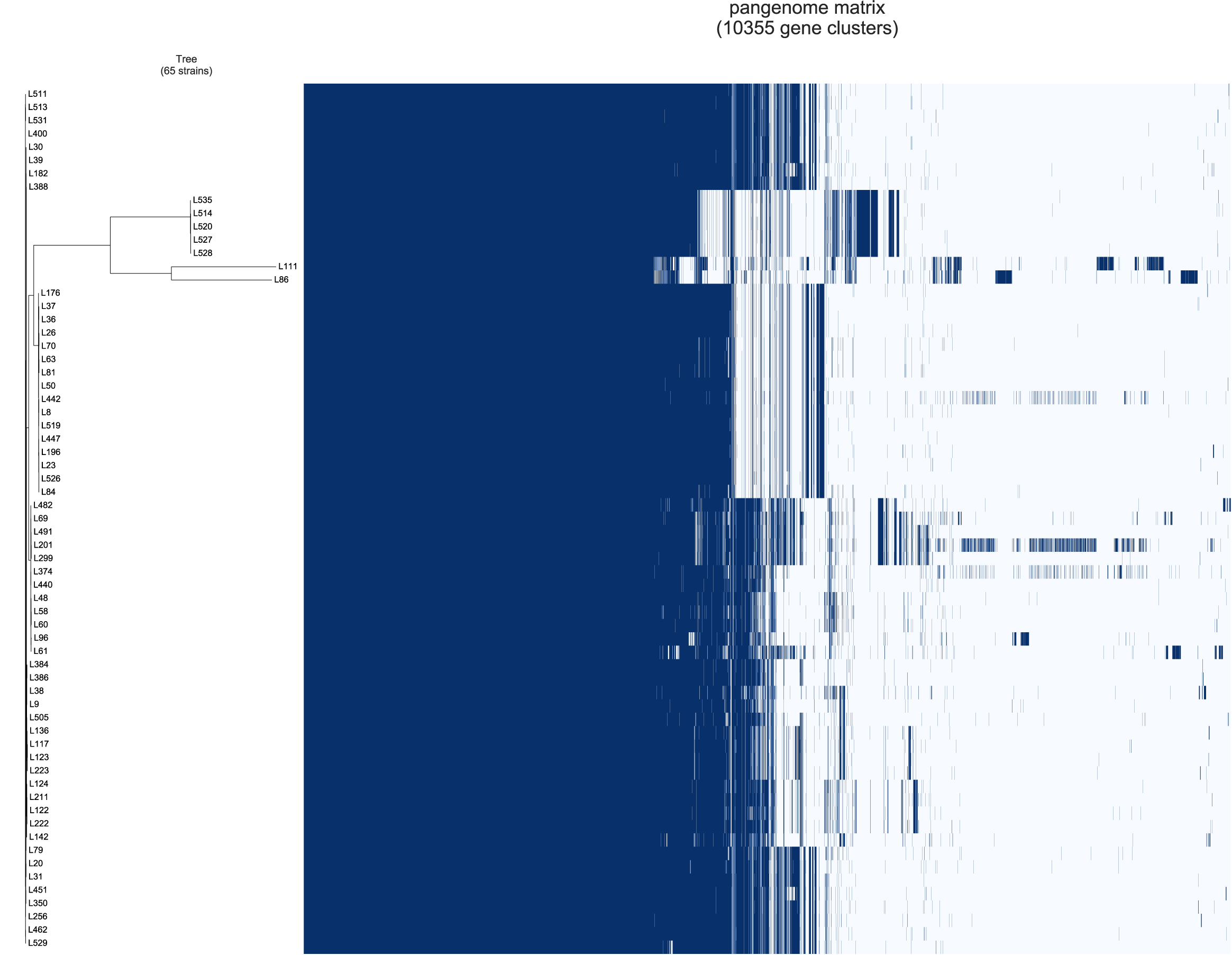

Supplement: FIG S4 [file mSystems.00498-20-sf004.tif]
