# Supplementary material for: Stool Samples of Acute Diarrhea Inpatients as a Reservoir of ST11 Hypervirulent KPC-2-Producing Klebsiella pneumoniae
Source: mSystems. 2020 Jun 23;5(3):e00498-20. doi: 10.1128/mSystems.00498-20 (PMC7311318; doi:10.1128/mSystems.00498-20)
Supplement: TABLE S1 [file mSystems.00498-20-st001.docx]

**Table S1**. Carbapenemase-producing *Enterobacteriaceae* isolates recovered from stool specimens.

| **Isolate** | **Species** | **Ward** | **Carbapenemase gene** | **Plasmid replicon typing** | **ST** ^a^ |
| --- | --- | --- | --- | --- | --- |
| L91 | *Citrobacter freundii* | Geriatric care | *bla*_IMP-4_ | NA | 98 |
| L5 | *Citrobacter koseri* | Gastroenterology | *bla*_KPC-2_ | IncFII, IncR | NA |
| L170 | *Citrobacter koseri* | ICU | *bla*_KPC-2_ | IncX4 | NA |
| L168 | *Citrobacter koseri* | ICU | *bla*_KPC-2_ | IncX4, IncFII | NA |
| L51 | *Enterobacter cloacae* | Gastroenterology | *bla*_IMP-26_ | IncHI2A | NA |
| L412 | *Escherichia coli* | ICU | *bla*_NDM-1_ | IncX4, IncFIB, IncN | 410 |
| L239 | *Escherichia coli* | Hepatobiliary & pancreatic surgery | *bla*_NDM-5_ | IncI1, IncB/O/K/Z, IncFII, IncX3 | 7454 |
| L214 | *Escherichia coli* | Hepatobiliary & pancreatic surgery | *bla*_NDM-5_ | IncI1, IncB/O/K/Z, IncFII, IncX3 | 7454 |
| L213 | *Escherichia coli* | Hematology | *bla*_NDM-5_ | IncI1, IncX3 | 617 |
| L200 | *Escherichia coli* | Bone marrow transplantation | *bla*_NDM-1_ | IncN, IncFII | 617 |
| L159 | *Escherichia coli* | Infectious diseases | *bla*_NDM-5_ | IncHI2, IncX3, IncFIB, IncN, IncX4 | 206 |
| L56 | *Klebsiella oxytoca* | ICU | *bla*_KPC-2_ | IncFIB | 43 |
| L47 | *Klebsiella oxytoca* | ICU | *bla*_KPC-2_ | IncFIB | 43 |
| L96 | *Klebsiella pneumoniae* | Hepatobiliary & pancreatic surgery | *bla*_KPC-2_ | IncR, IncX1, IncFII | 11 |
| L9 | *Klebsiella pneumoniae* | EICU | *bla*_KPC-2_ | IncFII | 11 |
| L86 | *Klebsiella pneumoniae* | Geriatric care | *bla*_KPC-2_ | IncFII, IncFIB, IncN | 107 |
| L84 | *Klebsiella pneumoniae* | ICU | *bla*_KPC-2_ | IncR | 11 |
| L81 | *Klebsiella pneumoniae* | ICU | *bla*_KPC-2_ | IncR | 11 |
| L8 | *Klebsiella pneumoniae* | ICU | *bla*_KPC-2_ | IncR | 11 |
| L79 | *Klebsiella pneumoniae* | Neurosurgery | *bla*_KPC-2_ | IncHI1B, IncR, IncFII | 11 |
| L70 | *Klebsiella pneumoniae* | ICU | *bla*_KPC-2_ | IncR, IncN | 11 |
| L69 | *Klebsiella pneumoniae* | Hepatobiliary & pancreatic surgery | *bla*_KPC-2_ | IncHI1B, IncI1, IncFII | 11 |
| L63 | *Klebsiella pneumoniae* | ICU | *bla*_KPC-2_ | IncR | 11 |
| L61 | *Klebsiella pneumoniae* | ICU | *bla*_KPC-2_ | IncHI1B, IncFII, IncFIB | 11 |
| L60 | *Klebsiella pneumoniae* | ICU | *bla*_KPC-2_ | IncFII, IncN, IncR | 11 |
| L58 | *Klebsiella pneumoniae* | ICU | *bla*_KPC-2_ | IncFII, IncN, IncR | 11 |
| L535 | *Klebsiella pneumoniae* | ICU | *bla*_KPC-2_ | IncFII, IncR, IncFIB | 37 |
| L531 | *Klebsiella pneumoniae* | ICU | *bla*_KPC-2_ | IncHI1B, IncR, IncFII | 11 |
| L529 | *Klebsiella pneumoniae* | Orthopaedic surgery | *bla*_KPC-2_ | IncHI1B, IncR, IncFII | 11 |
| L528 | *Klebsiella pneumoniae* | ICU | *bla*_KPC-2_ | IncFII, IncR, IncFIB | 37 |
| L527 | *Klebsiella pneumoniae* | Infectious diseases | *bla*_KPC-2_ | IncFII, IncR, IncFIB | 37 |
| L526 | *Klebsiella pneumoniae* | ICU | *bla*_KPC-2_ | IncR | 11 |
| L520 | *Klebsiella pneumoniae* | ICU | *bla*_KPC-2_ | IncFII, IncR, IncFIB | 37 |
| L519 | *Klebsiella pneumoniae* | Hepatobiliary & pancreatic surgery | *bla*_KPC-2_ | IncR | 11 |
| L514 | *Klebsiella pneumoniae* | Infectious diseases | *bla*_KPC-2_ | IncFII, IncR, IncFIB | 37 |
| L513 | *Klebsiella pneumoniae* | ICU | *bla*_KPC-2_ | IncHI1B, IncR, IncFII | 11 |
| L511 | *Klebsiella pneumoniae* | ICU | *bla*_KPC-2_ | IncHI1B, IncR, IncFII | 11 |
| L505 | *Klebsiella pneumoniae* | Urology | *bla*_KPC-2_ | IncR, IncFII | 11 |
| L50 | *Klebsiella pneumoniae* | Hepatobiliary & pancreatic surgery | *bla*_KPC-2_ | IncR | 11 |
| L491 | *Klebsiella pneumoniae* | EICU | *bla*_KPC-2_ | IncHI1B, IncI1, IncFII | 11 |
| L482 | *Klebsiella pneumoniae* | ICU | *bla*_KPC-2_ | IncHI1B, IncI1, IncFII | 11 |
| L48 | *Klebsiella pneumoniae* | ICU | *bla*_KPC-2_ | IncFII, IncN, IncR | 11 |
| L462 | *Klebsiella pneumoniae* | EICU | *bla*_KPC-2_ | IncHI1B, IncR, IncFII | 11 |
| L451 | *Klebsiella pneumoniae* | EICU | *bla*_KPC-2_ | IncHI1B, IncR, IncFII | 11 |
| L447 | *Klebsiella pneumoniae* | ICU | *bla*_KPC-2_ | IncR | 11 |
| L442 | *Klebsiella pneumoniae* | ICU | *bla*_KPC-2_ | IncR, IncN3 | 11 |
| L440 | *Klebsiella pneumoniae* | Infectious diseases | *bla*_KPC-2_ | IncR, IncFII | 11 |
| L400 | *Klebsiella pneumoniae* | ICU | *bla*_KPC-2_ | IncHI1B, IncR, IncFII | 11 |
| L39 | *Klebsiella pneumoniae* | EICU | *bla*_KPC-2_ | IncHI1B, IncR, IncFII | 11 |
| L388 | *Klebsiella pneumoniae* | Neurosurgery | *bla*_KPC-2_ | IncHI1B, IncR, IncFII | 11 |
| L386 | *Klebsiella pneumoniae* | Bone marrow transplantation | *bla*_KPC-2_ | IncR, IncFII | 11 |
| L384 | *Klebsiella pneumoniae* | Neurosurgery | *bla*_KPC-2_ | IncR, IncFII | 11 |
| L38 | *Klebsiella pneumoniae* | Infectious diseases | *bla*_KPC-2_ | IncFII, IncR | 11 |
| L374 | *Klebsiella pneumoniae* | Geriatric care | *bla*_KPC-2_ | IncR, IncFII | 11 |
| L37 | *Klebsiella pneumoniae* | Hepatobiliary & pancreatic surgery | *bla*_KPC-2_ | IncR | 11 |
| L36 | *Klebsiella pneumoniae* | ICU | *bla*_KPC-2_ | IncR | 11 |
| L350 | *Klebsiella pneumoniae* | Surgical ICU | *bla*_KPC-2_ | IncHI1B, IncR, IncFII | 11 |
| L31 | *Klebsiella pneumoniae* | ICU | *bla*_KPC-2_ | IncHI1B, IncR, IncFII | 11 |
| L30 | *Klebsiella pneumoniae* | Gastroenterology | *bla*_KPC-2_ | IncHI1B, IncR, IncFII | 11 |
| L299 | *Klebsiella pneumoniae* | ICU | *bla*_KPC-2_ | IncHI1B, IncI1, IncFII | 11 |
| L26 | *Klebsiella pneumoniae* | Hepatobiliary & pancreatic surgery | *bla*_KPC-2_ | IncR | 11 |
| L256 | *Klebsiella pneumoniae* | EICU | *bla*_KPC-2_ | IncHI1B, IncR, IncFII | 11 |
| L23 | *Klebsiella pneumoniae* | ICU | *bla*_KPC-2_ | IncR | 11 |
| L223 | *Klebsiella pneumoniae* | ICU | *bla*_KPC-2_ | IncR, IncFII | 11 |
| L222 | *Klebsiella pneumoniae* | Geriatric care | *bla*_KPC-2_ | IncFII, IncR, IncFIB | 11 |
| L211 | *Klebsiella pneumoniae* | Bone marrow transplantation | *bla*_KPC-2_ | IncFII, IncR, IncFIB | 11 |
| L201 | *Klebsiella pneumoniae* | EICU | *bla*_KPC-2_ | IncHI1B, IncI1, IncFII | 11 |
| L20 | *Klebsiella pneumoniae* | ICU | *bla*_KPC-2_ | IncHI1B, IncR, IncFII | 11 |
| L196 | *Klebsiella pneumoniae* | ICU | *bla*_KPC-2_ | IncR | 11 |
| L182 | *Klebsiella pneumoniae* | ICU | *bla*_KPC-2_ | IncHI1B, IncR, IncFII | 11 |
| L176 | *Klebsiella pneumoniae* | ICU | *bla*_KPC-2_ | IncR | 11 |
| L142 | *Klebsiella pneumoniae* | Surgical ICU | *bla*_KPC-2_ | IncR, IncFII | 11 |
| L136 | *Klebsiella pneumoniae* | ICU | *bla*_KPC-2_ | IncR, IncFII | 11 |
| L124 | *Klebsiella pneumoniae* | Respiratory diseases | *bla*_KPC-2_ | IncFII, IncR, IncFIB | 11 |
| L123 | *Klebsiella pneumoniae* | ICU | *bla*_KPC-2_ | IncR, IncFII | 11 |
| L122 | *Klebsiella pneumoniae* | ICU | *bla*_KPC-2_ | IncR, IncFII | 11 |
| L117 | *Klebsiella pneumoniae* | ICU | *bla*_KPC-2_ | IncR, IncFII | 11 |
| L111 | *Klebsiella pneumoniae* | Neurosurgery | *bla*_KPC-2_ | IncFIB, IncFII | 15 |
| L18 | *Klebsiella variicola* | Bone marrow transplantation | *bla*_KPC-2_ | IncP | NA |
| L241 | *Morganella morganii* | Surgical ICU | *bla*_NDM-5_ | NA | NA |
| L90 | *Proteus mirabilis* | Geriatric care | *bla*_KPC-2_ | IncN | NA |
| L76 | *Proteus mirabilis* | ICU | *bla*_KPC-2_ | IncN | NA |
| L71 | *Proteus mirabilis* | ICU | *bla*_KPC-2_ | IncN | NA |
| L52 | *Proteus mirabilis* | ICU | *bla*_KPC-2_ | IncN | NA |
| L49 | *Proteus mirabilis* | ICU | *bla*_KPC-2_ | IncN | NA |
| L44 | *Proteus mirabilis* | ICU | *bla*_KPC-2_ | IncN | NA |
| L59 | *Raoultella ornithinolytica* | Infectious diseases | *bla*_IMP-4_ | IncFIB, IncU | NA |

^a^ NA, not available
