# Supplementary material for: Stool Samples of Acute Diarrhea Inpatients as a Reservoir of ST11 Hypervirulent KPC-2-Producing Klebsiella pneumoniae
Source: mSystems. 2020 Jun 23;5(3):e00498-20. doi: 10.1128/mSystems.00498-20 (PMC7311318; doi:10.1128/mSystems.00498-20)
Supplement: TABLE S2 [file mSystems.00498-20-st002.docx]

**Table S2**. Summary of the carbapenemase-producing Enterobacteriaceae (CPE) isolates and the β-lactamases encoded by the CPE isolates.

|  |  | **Carbapenemases** | | | | | **ESBLs** | |
| --- | --- | --- | --- | --- | --- | --- | --- | --- |
| **Species** | **Number** | **IMP-4** | **IMP-26** | **KPC-2** | **NDM-1** | **NDM-5** | **CTX-M-1 group** | **CTX-M-9 group** |
| *Citrobacter freundii* | 1 (1.1) | 1 | - | - | - | - | - | - |
| *Citrobacter koseri* | 3 (3.4) | - | - | 3 | - | - | - | 3 |
| *Enterobacter cloacae* | 1 (1.1) | - | 1 | - | - | - | - | - |
| *Escherichia coli* | 6 (6.9) | - | - | - | 2 | 4 | 1 | 1 |
| *Klebsiella variicola* | 1 (1.1) | - | - | 1 | - | - | - | - |
| *Klebsiella oxytoca* | 2 (2.3) | - | - | 2 | - | - | - | - |
| *Klebsiella pneumoniae* | 65 (74.7) | - | - | 65 | - | - | 4 | 39 |
| *Morganella morganii* | 1 (1.1) | - | - | - | - | 1 | - | - |
| *Proteus mirabilis* | 6 (6.9) | - | - | 6 | - | - | - | - |
| *Raoultella ornithinolytica* | 1 (1.1) | 1 | - | - | - | - | 1 | - |
| Total | 87 (100) | 2 | 1 | 77 | 2 | 5 | 6 | 43 |
